# Using the ‘Twelve tips for applying change models’ for undergraduate medical curriculum reform in Pakistan: Incorporating a new Trauma Evaluation and Management TEAM® course

**DOI:** 10.12688/mep.17507.2

**Published:** 2022-08-05

**Authors:** Rufina Soomro, Sheeraz Ur Rehman, Sobia Ali, Judy McKimm

**Affiliations:** 1 General Surgery and Health Care Education, Liaquat National Hospital and Medical College, Karachi, Sind, 74800, Pakistan; 2 Department of Health Professions Education, Liaquat National Hospital and Medical College, Karachi, Sind, 74800, Pakistan; 3 Department of Medical Education, Swansea University Medical School/Ysgol Meddygaeth, Swansea University, Prifysgol Abertawe, Swansea, Abertawe, Wales, Cymru, SA2 8PP, UK

**Keywords:** Curriculum design, TEAM, Curriculum change

## Abstract

**Background: **Trauma evaluation and management skills are not taught enough in medical school undergraduate curriculums worldwide. It has been recommended by trauma educators to incorporate trauma training in medical schools’ curriculum as first-line management of trauma cases is usually required by junior doctors in ERs. The introduction of formal trauma training in the form of the Trauma Evaluation and Management TEAM® course is a change introduced into the curriculum. Even when introducing such a simple change, certain factors need to be considered including the stakeholders’ apprehensions and involvement, the complexity of the internal and external environment, cultural context and political influences, and finally the psychological impact of change.

**Methods: **Based primarily on the *“* *Twelve tips for applying change models to curriculum design, development and delivery”* by McKimm and Jones (2018), these 12 tips provide educators, involved in curriculum or program development, a practical example of the systematic and organized outlines to improve medical curricula.

**Results & Conclusions: **While addressing these factors, this framework can guide educators for the successful development and implementation of a suggested change in the existing curriculum.

## Introduction

Curricular change in medical education can be a daunting process ( Ali & Baig, 2012). Curricular review, modification, and renewal are required regularly to meet the ever-growing demands of a modern curriculum. Developing countries, such as Pakistan, are constantly faced with the challenge of updating their curricula in line with national health and workforce needs and international standards ( Hamad, 1999; Magzoub & Schmidt, 1998). According to Bland, “successful curricular change occurs only through the dedicated efforts of effective change agents” ( Bland *et al.*, 2000). However, this often requires juggling priorities and competing stakeholder demands to strike the right balance. Curriculum change agents can face many setbacks, but effective curriculum reform can be very rewarding.

Trauma has been identified as a global issue and teaching undergraduates to manage trauma treatment effectively is challenging. Evidence suggests that trauma evaluation and treatment skills are under-represented in the medical school undergraduate curriculum around the world, and junior doctors are usually first responders who are required to manage severe trauma cases on their own. A study by Riaz *et al.* (2020) concluded that undergraduate medical students are exposed to trauma patients during their surgical clerkships, but that there is no structured or systematic trauma training curriculum in Pakistan. One way to counter these challenges is to produce competent/resilient doctors who can address challenging health and social situations and improve the health of the population. This requires a robust undergraduate medical curriculum that helps support future doctors

There is a paucity of evidence investigating the necessity for the implementation of a structured trauma course in the existing medical school curriculum in general and also in Pakistani literature ( Hoyler *et al.*, 2015; Hyder *et al.*, 2017; and Puvanachandra *et al.*, 2015). With the necessity for proper trauma training in mind, Liaquat National Hospital & Medical College (LNH&MC) chose to teach a Trauma Evaluation and Management (TEAM®) to its fourth year Bachelor of Medicine and Surgery (MBBS) students in their Orthopedic and Trauma Module.

In this article, we share our experiences of incorporating a TEAM® course into our existing undergraduate medical curriculum using a framework of twelve tips provided by McKimm & Jones (2018). We aim to provide educators with a practical example of using such frameworks to improve medical curricula.

## Tip #1

### Identify the purpose and scope of change

Trauma is a leading cause of death worldwide, especially in people younger than 44 years of age ( CDC, 2018). World Health Organization (WHO) figures suggest that more than nine people die every minute because of trauma and 5.8 million people die every year, irrespective of their age or economic status, due to accidental injuries. This accounts for 18% of the world’s total disease burden ( World Health Organization, 2013). Road traffic accidents are the most common type of trauma accounting for 1.2 million deaths per year and 20 to 50 million people sustain significant injuries. Whilst more than 90% of the road traffic crashes happen in low and middle-income countries (LMICs), they possess only 48% of the world’s motor vehicles. By the year 2030, Injury-related mortality in LMICs are anticipated to rise by 80% compared to current levels, due to inadequate safety measures ( World Health Organization, 2010).

A survey in Pakistan identified road traffic accidents as one of the major causes of injury with an annual incidence of 15 injuries per 1000 people ( Ghaffar *et al.*, 2004). Injuries in children are commonly seen more in incidences involving less educated mothers ( Singer & Ghaffar, 2004). Apart from unintentional injuries, other intentional injuries and violence are common. The rate of violence-related deaths in LMICs as a whole is more than twice that in high-income countries ( Krug *et al.*, 2002). Domestic violence ( Watts & Zimmerman, 2002), blast injuries, and firearm injuries are not uncommon ( Nasrullah & Razzak, 2009). Gunshots are seen more in young male civilians because of disputes ( Bukhari, 2017) and suicides. The mortality rates for seriously injured victims are six times higher in low-income countries than in the level 1 trauma centers in the US ( Mock *et al.*, 1993). Acute trauma patients can be managed with simple, easy-to-learn techniques, thus minimizing the mortality and morbidity.

Even in the US, 2.47% of trauma victims' deaths are due to medical errors, mainly failure to intubate and secure the airway, inappropriate hemorrhage control, and inadequate handling of seriously injured trauma victims ( Gruen *et al.*, 2006). In Pakistan, the rate is likely to be much higher because accident and emergency room staff in Pakistan are not well trained in trauma management and there is a lack of facilities to deal with serious trauma patients. There isn't enough emphasis on trauma evaluation and management skills in medical school curriculum in Pakistan. However, it is important to note that these are actually done by junior physicians & surgeons in majority of the cases. Riaz *et al.*. (2020).

 In trauma cases, the sooner specialized care can be provided the better the outcomes will be. However, in Pakistan, pre-hospital care and ambulance transport systems are relatively underdeveloped ( Minhas *et al.*, 2014). Also, the concept of managing trauma victims even in large hospitals has not been given enough emphasis through formal education with most trauma doctors learning experientially, which is often at the cost of lives. 

Advance Trauma Life Support (ATLS®) has now been introduced in more than 80 countries and it has demonstrated improved trauma care where it is practiced ( Abu-Zidan, 2016). Presently, the ATLS courses offered in Pakistan are optional and are intended only for health care professionals in specialized trauma units or postgraduate training.

Considering these issues, it was felt that if structured trauma teaching could be provided at the undergraduate level, graduating doctors will be able to provide better trauma care. The American College of Surgeons Committee on Trauma (ACS COT) developed the TEAM® course for medical students to address the challenges with ATLS training for medical students. TEAM® is a shorter version of ATLS designed for medical students as an introduction to trauma care. It has improved trauma teaching in undergraduate medical students in several developed and developing countries ( Ali *et al.*, 2004; Hill *et al.*, 2018; Lum & Subramaniam, 2016; and Soomro & Ali, 2020)

## Tip #2

### Create the vision, aligned to the mission

LNH&MC was founded with the vision of offering an unparalleled learning experience in health sciences with a resolve to produce ethical and dedicated professionals who will deliver evidence-based quality healthcare globally, and produce a visible impact on the health of the communities they serve.

Considering the College’s mission and vision to have relevant community-based teaching which positively impacts communities, trauma was identified as a serious area where timely medical intervention can save lives. There is a need to incorporate a comprehensive trauma management course where students have a theoretical understanding as well as practical experience in diagnosing and managing trauma victims, where simple maneuvers/interventions can save a life, reduce medical errors, and morbidity. To incorporate such a comprehensive trauma course, within the existing curriculum, further planning was needed and a need for a locally oriented trauma course was identified. The proposed course had to meet national and international requirements for providing ethically centered health care with compassion and dedication, as well as cultural understanding.

## Tip #3

### Develop a strategy for change involving key stakeholders

To achieve the change, it was crucial to identify the key leader/course director who would provide the framework for collaborative leadership and ensure that all stakeholders are involved and represented in the change process ( Table 1). It was important to keep the institutional cultures and reporting relationships in mind to avoid potential conflict.

**Table 1.** Key Stakeholders.

a. ACS Trauma Division, USA b. Curriculum Committee/College Administration c. Dean Medical College d. Department of Health Professions Education e. Course Director f. Faculty Members g. Hospital Administration h. Medical Students i. Simulated Patients

Once the leader was identified, a ‘guiding coalition’ was formed of like-minded faculty members which included mostly ATLS certified instructors and providers and members of the Department of Health Professions Education (DHPE), who had a good understanding of the importance of teaching trauma to undergraduate students. The strategy also involved identifying existing resources, so that the senior leadership of the College could be convinced that conducting such a course is possible.

Suitable teaching methodologies were identified through a literature search and the TEAM® course was selected based on the available resources. Once faculty members had agreed that it was feasible for the course to run within the existing curriculum, to gain formal approval, a proposal was forwarded to the competent authorities, including the medical college curriculum committee and hospital administration.

After their approval, the course director approached the ACS Trauma division to seek permission for the course to be conducted at the College.

## Tip #4

### Quick visible wins and communications are vital

Based on years of organizational investigation, Kotter ( Kotter, 1996) outlined the eight-stage transformational framework in greater depth. Many of the steps are highlighted in this framework for sustainable change: establishing a feeling of urgency, forming a strong steering coalition, the importance of short-term wins, the process of consolidating changes, and thus achieving additional change. Relying on Kotter’s suggestion, we believe that as curricular change takes a long time and if quick wins are not planned, inertia starts to set in, leading to a loss of momentum. For this, we actively planned to create short-term wins in terms of the dissemination of faculty and learners’ positive experiences of the TEAM® course. Along with a letter of thanks from the course director, a certificate of appreciation was also distributed to all faculty involved in TEAM® course planning and implementation. Participating students were also awarded certificates of attendance with the logo of the American College of Surgeons (with permission) and Liaquat National Hospital & Medical College. These certificates were distributed in a ceremony attended by the senior management of the college and hospital, along with students and faculty. In addition to a short presentation about the TEAM® course design, structure, its implementation process, and assessment, student and faculty feedback were also shared. This helped faculty not involved in TEAM® planning or implementation to start visualizing a successful curricular change model and the essential components that need to be addressed to achieve positive change. Hence, this also helped the transformation effort to slowly take root in the culture of the organization.

A comprehensive TEAM® report was also developed which was forwarded to the curriculum committee and TEAM® course developer at the ACS. This report thus became a vital communication resource in addition to the planning and evaluation of teaching, learning, and assessment. The following elements are among the most important components of the report:

1. Objectives
2. Identification of target audience and key stakeholders,
3. Rules and policies to be followed by students and faculty,
4. Course outline,
5. Schedule of the workshop along with pre and post-tests schedule,
6. Stations’ descriptions,
7. Teaching-learning strategies
8. Assessment plan
9. Resource requirement
10. List of Students with pre & post-test results
11. List of Faculty with credentials
12. Summary of Student Feedback
13. Summary of Faculty Feedback
14. Copy of the certificates for faculty and students
15. Snapshots of Skills Stations

## Tip #5

### Analyze the internal environment and culture

To develop a sustainable curriculum, the external and internal environments should be analyzed for strengths and weaknesses. A SWOT analysis ( D’Anselme *et al.*, 2020) helped to provide insight into the internal environment. At the heart of the internal environment lies the ‘cultural web’ ( Doherty & Stephens, 2020) which incorporates the people involved (students, faculty, and administrative staff), requirements and expectations from the curriculum, teaching strategies, ways of delivering the curriculum, learning spaces, and funding.

The three facets (students, faculty and administrations) had shared values and expectations (in terms of enhanced trauma management skills). These values provided a conducive internal environment that offered minimal resistance and easy rollout e.g. having ATLS certified faculty who were keen to teach, accommodating administration willing to support the change leader’s idea of innovation and students perspective of learning enhance trauma management protocols at a relatively early stage.

It is also of paramount importance the change leader should acknowledge everyone’s perspective and it is useful to step back and ‘re-frame’ the entire process in light of the political, symbolic, organizational values, goals and objectives, and structural processes involved ( Tierney, 1988).

Based on the internal analyses, it was decided that there would be a ‘re-brand’ of the relevant parts of the curriculum at intervals to incorporate feedback from all stakeholders, ensure sustainability, and reduce negativity. The following strategies taken by the course director proved helpful:

1. Use of the logo of the American College of Surgeons® in the certification process
2. The formal introduction of the new curriculum in collaboration with the Department of Health Professions Education (DHPE) and ATLS® certified instructors and providers. This strategy promoted the strength of faculty to deliver the course.
3. Formal acknowledgment of all stakeholders through seminars.
4. Dissemination of post-hoc results for consumption by the relevant stakeholders.
5. Regular feedback from the students and making swift changes in the program to ensure sustainability.

## Tip #6

### Consider the external environment, cultural contexts, and political influences

Just focusing on the internal environment and disregarding the external environment could seriously jeopardize curriculum stability. The framework ( Bush, 2016) which involves the assessment of political, economic, socio-cultural, technological, legal, and environmental factors (PESTLE), can help in evaluating the revised curriculum in light of external regulatory bodies.

In this context, undertaking a PESTLE analysis was very helpful since the College’s affiliation with Jinnah Sindh Medical University (J.S.M.U) and the Pakistan Medical and Dental Council (PMDC) could lead to unintended consequences and potentially threaten the curriculum. Running the TEAM® course is intense, requiring a strong commitment from all stakeholders. Since none of the affiliated colleges with the J.S.M.U were offering this course and were using traditional teaching methods to teach trauma, it was important to convince them that the change is sustainable and reproducible at regional and national levels.

Since the College has always been a front runner in many innovative teaching programs, the change leaders were determined to run the course and continue the legacy. Sustainability was ensured through regular program evaluations and sharing results with external bodies. Regulatory bodies such as the PMDC look for examples of innovation and this is where the TEAM® course stood out.

## Tip #7

### Choose the right combination of approaches to change

A recommendation by McKimm & Jones (2018) was used as an example of how to achieve a balance between certain outcomes, interests and emotions. The introduction of TEAM® to the fourth year MBBS students was based on the rationale that:

1. The outcomes of the TEAM® course can be aligned with the outcomes of the Orthopedics and Trauma Module offered to the same year by the affiliated University ( JSMU, 2020)

2. Orthopedics and ER rotations are mandatory for the fourth year MBBS students and knowledge and skills gained during this course would help strengthen the clinical curricula in both departments. 

3. Implementing TEAM® for medical students has better outcomes in terms of knowledge and skill outcomes ( Delgado-Reyes *et al.*, 2016), ( Cherry *et al.*, 2005).

In addition to curriculum alignment, the course director met with each member of the course TEAM® so that all had a clear idea of their role considering their competencies and experience. Teaching faculty were assigned stations according to their specialty (for example, neurosurgeons were stationed at helmet removal and application of cervical collar and faculty from ER were on the triage station). Staff from the skill labs were involved in resource provision (including the venue, mannequins, audiovisual (AV) aids, and instruments) and faculty from DHPE supported educational activities including station development, assessment, and evaluation. The interest of each member was therefore maintained as they were assigned tasks that best suit their capabilities. 

## Tip #8

### Use project management techniques for operational planning and implementation

When the TEAM® course was planned, many issues were considered: available resources, course materials, financial support, and support of curriculum committee/DHPE, venue, timelines, and placement in the curriculum, and types of assessment. Essential change management techniques are necessary to bring desired results. We adopted Kurt Lewin’s change model (unfreeze, changing and refreeze), since it is best applied in socio-cultural context of our organizational environment ( Hussain *et al.*, 2018).

Accumulating all these elements was an uphill task and a project management approach greatly helped to identify all the details and critical paths, for example:

1. Ground rules were set for students and faculty, relating to discipline and punctuality.
2. Copyrighted materials were purchased for every student from ACS. Books and DVDs were distributed to students at the beginning of the module.
3. TEAM® faculty were recruited and selected comprising the Course Director, ATLS instructor, and other ATLS trained faculty members in surgical and Allied discipline and ER along with Representation from Department of Health Professions Education.
4. Multiple choice questions (MCQs) were used for formative assessment and had a pre-post design. The examination paper was reviewed and approved by the International ATLS Educator.
5. With permission from the curriculum committee and discussion with the faculty, the TEAM® course was included as part of the “Orthopedic and Trauma” module.
6. The course was slightly modified to the context after discussing it with the International ATLS® educator. Various instructional strategies such as interactive lectures, small group discussions, video tutorials with good and bad versions, and skills lab sessions were used to give an enriched experience. A special focus was on skills development and stimulating deep learning, using case-based small group discussions and simulated patients.
7. Time management was a challenge, so the concept of Teaching Objective Structured Clinical Examination (TOSCE) ( Amini *et al.,* 2012) was used. Six stations were staffed by two certified/trained instructors with four-five students per station with a 30-minute encounter time. On-site feedback was provided at each station.

Although no formal project management software was used, employing the principles of project management greatly improved efficiency and effectiveness.

## Tip #9

### Acknowledge the psychological impact of change

In the excitement of introducing a new course into an existing curriculum, we tried not to underestimate the possible resistance or reluctance of stakeholders. Even a little modification in a curriculum can create repercussions among faculty due to increased job demands and decreased job control. The TEAM® course at LNH&MC was offered in the form of a five-hour workshop for four consecutive Thursdays and it was recognized that asking busy clinicians for this extra time commitment may cause stress in terms of increased job demands. Similarly, faculty that are used to providing lectures independently were required to work in pairs at each station. Therefore, there may have been increased anxiety due to role conflict or feeling under scrutiny, and this can lead to feelings of decreased job control.

The course director’s meetings with faculty before the implementation of the course helped to address faculty’s concerns, reduce anxiety, clarify the outcomes, address their own as well as each members’ limitations, disseminate measures for successful implementation and, most importantly, make collaborative decisions ( Armstrong & Taylor, 2020).

## Tip #10

### Plan for transition and loss of competence

There are many challenges in implementing a new course into an established curriculum.

When the TEAM® course began, faculty were asked to move out of their comfort zone of traditional teaching to a more student-centered approach that involved using mannequins and simulated patients in small groups. To overcome this daunting task, the faculty were given appropriate support and feedback. For each station, separate sessions were conducted under the supervision of the course director to ensure each faculty member could facilitate learning and assess the students. At every station, each faculty member was supported by another competent co-faculty member, and they were given extra time to prepare for this new role.

The students were also not used to this way of learning and the pre-post assessment design hence requiring support and reassurance in the form of introductory sessions, constructive feedback during the learning process, and group discussion and activities.

Bringing this change was not easy and only possible when all stakeholders were satisfied. The Course Director, DHPE, and the college administration’s huge efforts contributed to the success of the change.

## Tip #11

### Don’t underestimate the complexity

The TEAM® course is a patented course of the ACS that provides all the material including a faculty manual, slide presentations, skills videos, and a student manual and as such, ATLS certified faculty usually find the course easy to run.

Organizing the course is complex, however, because trauma has no anatomical boundaries, so all the surgical and allied disciplines, as well as those from the emergency room are involved. A large faculty pool is needed as the duration of this course is five hours over a few weeks and busy clinical teachers may not be available for that length of time. A large contribution is also needed from the faculty of DHPE and Skills laboratory. In this interconnected system with many players involved, it might be hard to stop deviating from the intended goals, it is, therefore, important to have good adaptive leadership to keep everyone involved and motivated in working towards the shared aims. 

The TEAM® course is well-structured and has a lot of room for flexibility. This led to the need for local decisions by the task force in collaboration with all stakeholders such as the number of stations, their content, and teaching methods including skill acquisition, discussions, and patient management.

In addition to the explicit formal curriculum, we realized that there was an implicit curriculum running alongside. In response to student queries and concerns, the course director and medical educators adapted the course to add more models and content around patient assessment and management and more explanations about their use. 

## Tip #12

### Celebrate success and the shift from project to “ *new reality*”

Whether introducing a new course in an existing curriculum or transforming the whole curriculum and its approach, the change should not be considered a destination, but a carefully designed process. Many change initiatives fail to achieve reality, therefore a deeper understanding of approaches and how to address challenges to alleviate risk is mandatory. A carefully structured plan before introducing the TEAM® course into an existing module helped convert an idea into actuality. After identifying and addressing the findings from a SWOT analysis (Tip 6 & 7) and keeping all the stakeholders on board, the course was formally launched via an introductory seminar that discussed the importance, the structure, and the process of working during the course. Faculty, students, College and Hospital Administration, Skills Lab staff, members of the curriculum committee, and faculty of the DHPE attended the seminar. A certificate distribution ceremony for students and faculty also helped in celebrating ‘quick wins’ (Tip 4). 

## Conclusion

The successful adoption of a new course in an established curriculum necessitates extensive planning. This comprises not just operational planning for stakeholders’ involvement, organizational culture analysis, and execution plans but also post-implementation monitoring measures for curriculum alignment. Given the multifaceted nature of curriculum development and implementation, educators require practical tips and resources that assist them to deal with this complexity. These twelve tips share our first-hand experience of curricular change implementation, from simple project planning and delivery to complicated organizational culture and stakeholders’ management handling. Our focus is on providing practical tools that educators, managers, and administrators can use to reform and review the high-quality change in educational programs. We expect that others may benefit from thinking forward and planning for the intricacies of curriculum reform to prevent mistakes and achieve their goals.

## Data availability

### Underlying data

EUDAT: Using the ‘Twelve tips for applying change models’ for undergraduate medical curriculum reform in Pakistan: Incorporating a new Trauma Evaluation and Management TEAM® course. http://doi.org/10.23728/b2share.702dcbf1b6874a289ba137a7db3ce10a ( Soomro *et al.*, 2022)

This project contains the following underlying data:

- TEAM® data for the 12 Tips.rar

### Extended data

This project contains the following extended data:

- List of documents submitted for 12 TIPs TEAM®.docx

Data are available under the terms of the Creative Commons Zero "No rights reserved" data waiver (CC0 1.0 Public domain dedication).

## References

[ref-2] Abu-Zidan FM : Advanced trauma life support training: How useful it is? *World J Crit Care Med.* 2016;5(1):12–6. 10.5492/wjccm.v5.i1.12 26855889 PMC4733451

[ref-3] Ali J Danne P McColl G : Assessment of the trauma evaluation and management (TEAM) module in Australia. *Injury.* 2004;35(8):753–758. 10.1016/j.injury.2003.10.023 15246797

[ref-4] Ali SK Baig LA : Problems and issues in implementing innovative curriculum in the developing countries: the Pakistani experience. *BMC Med Educ.* 2012;12:31. 10.1186/1472-6920-12-31 22591729 PMC3395573

[ref-5] Amini M Moghadami M Kojuri J : Using TOSCE (Team Objective Structured Clinical Examination) in the second national medical sciences olympiad in Iran. *J Res Med Sci.* 2012;17(10):975–8. 23826000 PMC3698659

[ref-6] Armstrong M Taylor S : Armstrong's handbook of human resource management practice.Kogan Page Publishers, 2020. Reference Source

[ref-7] Bland CJ Starnaman S Wersal L : Curricular change in medical schools: how to succeed. *Acad Med.* 2000;75(6):575–94. 10.1097/00001888-200006000-00006 10875502

[ref-8] Bukhari A : Distribution of firearm injuries presented to the surgical department of DHQ teaching hospital Bannu, Pakistan. *Gomal Journal of Medical Sciences.* 2017;15(2). Reference Source

[ref-9] Bush T : PESTLE Analysis: Everything You Need to Know.PESTLE Analysis. 2016. Reference Source

[ref-10] CDC: Leading Causes of Death and Injury.Online: Centers for Disease Control and Prevention, National Center for Injury Prevention and Control. 2018; [Accessed December 11, 2020]. Reference Source

[ref-11] Cherry RA Ali J Williams JI : Trauma evaluation and management: who benefits among medical students? *J Surg Res.* 2005;126(2):189–92. 10.1016/j.jss.2005.02.001 15919418

[ref-12] d’Anselme O Pelligand L Veres-Nyeki K : Analysis of teaching methods in anaesthesia in the undergraduate curriculum of four veterinary universities. *Vet Anaesth Analg.* 2020;47(5):657–666. 10.1016/j.vaa.2020.02.011 32792273

[ref-13] Delgado-Reyes L Gasca-González OO Delgado-Guerrero F : [Effectiveness of Trauma Evaluation and Management course for Mexican senior medical students: When to implement it?] *Cirugía y Cirujanos (English Edition).* 2016;84( *3*):220–224. 10.1016/j.circen.2016.04.004 26738652

[ref-14] Doherty O Stephens S : The cultural web, higher education and work-based learning. *Industry and Higher Education.* 2020;34(5):330–341. 10.1177/0950422219879614

[ref-16] Ghaffar A Hyder AA Masud TI : The burden of road traffic injuries in developing countries: the 1st national injury survey of Pakistan. *Public Health.* 2004;118(3):211–7. 10.1016/j.puhe.2003.05.003 15003410

[ref-17] Gruen RL Jurkovich GJ McIntyre LK : Patterns of errors contributing to trauma mortality: lessons learned from 2,594 deaths. *Ann Surg.* 2006;244(3):371–80. 10.1097/01.sla.0000234655.83517.56 16926563 PMC1856538

[ref-18] Hamad B : Establishing community‐orientated medical schools: key issues and steps in early planning. *Med Educ.* 1999;33(5):382–389. 10.1046/j.1365-2923.1999.00287.x 10336775

[ref-19] Hill KA Johnson ED Lutomia M : Implementing the Trauma Evaluation and Management (TEAM) Course in Kenya. *J Surg Res.* 2018;232:107–112. 10.1016/j.jss.2018.05.066 30463705

[ref-20] Hoyler M Hagander L Gillies R : Surgical care by non-surgeons in low-income and middle-income countries: a systematic review. *Lancet.* 2015;385 Suppl 2:S42. 10.1016/S0140-6736(15)60837-6 26313091

[ref-40] Hussain ST Lei S Akram T : Kurt Lewin's change model: A critical review of the role of leadership and employee involvement in organizational change. *J Innov Knowl.* 2018;3(3):123–127. 10.1016/j.jik.2016.07.002

[ref-21] Hyder AA He S Zafar W : One hundred injured patients a day: multicenter emergency room surveillance of trauma in Pakistan. *Public Health.* 2017;148:88–95. 10.1016/j.puhe.2017.03.006 28431334

[ref-1] Jinnah Sind Medical University study guide MBBS 2020. JSMU, 2020; [Accessed 30/11/2020 2020].

[ref-22] Kotter JP : Leading Change.Harvard Business School Press, Boston, MA,1996. Reference Source

[ref-23] Krug EG Mercy JA Dahlberg LL : The world report on violence and health. *Lancet.* 2002;360(9339):1083–1088. 10.1016/S0140-6736(02)11133-0 12384003

[ref-24] Lum SK Subramaniam T : The teaching of trauma management in undergraduate medical education. *Med J Malaysia.* 2016;71(6):338–340. 28087958

[ref-25] Magzoub ME Schmidt HG : Testing a causal model of community-based education in the Sudan. *Acad Med.* 1998;73(7):797–802. 10.1097/00001888-199807000-00021 9679471

[ref-26] McKimm J Jones PK : Twelve tips for applying change models to curriculum design, development and delivery. *Med Teach.* 2018;40(5):520–526. 10.1080/0142159X.2017.1391377 29069976

[ref-27] Minhas MS Khan KM Effendi J : Improvised explosive device bombing police bus: Pattern of injuries, patho-physiology and early management. *J Pak Med Assoc.* 2014;64(12 Suppl 2):S49–S53. 25989781

[ref-28] Mock CN Adzotor KE Conklin E : Trauma outcomes in the rural developing world: comparison with an urban level I trauma center. *J Trauma.* 1993;35(4):518–23. 10.1097/00005373-199310000-00004 8411273

[ref-29] Nasrullah M Razzak JA : Firearm injuries presenting to a tertiary care hospital of Karachi, Pakistan. *J Inj Violence Res.* 2009;1(1):27–31. 10.5249/jivr.v1i1.27 21483188 PMC3134905

[ref-33] Puvanachandra P Razzak JA Hyder AA : Establishing a National Emergency Department Surveillance: an innovative study from Pakistan. *BMC Emerg Med.* 2015;15 Suppl 2(Suppl 2):I1. 10.1186/1471-227X-15-S2-I1 26690384 PMC4682373

[ref-34] Riaz Q Saqib SU Siddiqui NA : Changing face of trauma and surgical training in a developing country: A literature review. *J Pak Med Assoc.* The Journal of the Pakistan Medical Association,2020;70(Suppl 1)(2):S89–S94. 31981343

[ref-35] Singer MS Ghaffar A : Risk factors for road traffic injury in Pakistani children. *J Coll Physicians Surg Pak.* 2004;14(12):709–12. 15610626

[ref-36] Soomro R Ali S : Trauma Evaluation and Management TEAM® course for medical students in Pakistan. *Pak J Med Sci.* 2020;36(6):1257–1262. 10.12669/pjms.36.6.2588 32968390 PMC7501016

[ref-37] Soomro R Rehman S Ali S : B2SHARE. Supplementary Data 12 TIPS.2022; [Accessed 14 March 2022]. 10.23728/b2share.702dcbf1b6874a289ba137a7db3ce10a

[ref-38] Tierney WG : Organizational culture in higher education: Defining the essentials. *J High Educ.* 1988;59(1):2–21. 10.2307/1981868

[ref-39] Watts C Zimmerman C : Violence against women: global scope and magnitude. *Lancet.* 2002;359(9313):1232–7. 10.1016/S0140-6736(02)08221-1 11955557

[ref-30] World Health Organization: Injuries and violence: the facts.World Health Organization,2010. Reference Source

[ref-31] World Health Organization: Global status report on road safety 2013: supporting a decade of action: summary.World Health Organization,2013. Reference Source

