# Supplementary material for: Using the ‘Twelve tips for applying change models’ for undergraduate medical curriculum reform in Pakistan: Incorporating a new Trauma Evaluation and Management TEAM® course
Source: MedEdPublish (2016). 2022 Aug 5;12:29. Originally published 2022 Apr 22. [Version 2] doi: 10.12688/mep.17507.2 (PMC9926506; doi:10.12688/mep.17507.2)
Supplement: Peer review history

# Peer review: Reviewer response for version 2

**DOI:** 10.21956/mep.20603.r32796

**Published:** 2022-12-23

**Authors:** Susan Camille van Schalkwyk

**Affiliations:** 1 Centre for Health Professions Education, Faculty of Medicine and Health Sciences, Stellenbosch University, Stellenbosch, South Africa

Thank you for attending to the comments made on the previous version of this paper. I believe that the changes further enhance the focus of the work.

If evidence from practice is presented, are all the underlying source data available to ensure full reproducibility?

Yes

Is the topic of the practical tips discussed accurately in the context of the current literature

Partly

Are all factual statements correct and adequately supported by citations?

Partly

Are the conclusions drawn balanced and justified on the basis of the presented arguments?

Partly

Are arguments sufficiently supported by evidence from the published literature and/or the authors’ practice?

Partly

Reviewer Expertise:

In the context of this article: curriculum renewal

I confirm that I have read this submission and believe that I have an appropriate level of expertise to confirm that it is of an acceptable scientific standard.

# Peer review: Reviewer response for version 1

**DOI:** 10.21956/mep.18782.r31897

**Published:** 2022-05-20

**Authors:** Susan Camille van Schalkwyk

**Affiliations:** 1 Centre for Health Professions Education, Faculty of Medicine and Health Sciences, Stellenbosch University, Stellenbosch, South Africa

This review is based on the version of the article first published on 22 April 2022.

 This article makes use of the framework (12 Tips) published by McKimm and Jones (2018). Its focus is an important one – that of effecting curriculum change – and it documents an institutional intervention to introduce a structured trauma course into an existing curriculum drawing on and ‘slightly modifying’ an existing course from elsewhere in the world. The intention of the authors is to provide a set of practical tips for others embarking on a similar initiative. The ‘TEAM’ course that is described sounds interesting. The authors describe it as ‘well-structured’ and ‘flexible’ which it does indeed appear to be. I did, however, wonder about the extent to which the provision of these ‘tips’ will have wider applicability. Trauma training requires our overt attention, particularly in low and middle-income countries where the numbers of deaths through motor vehicle accidents, for example, are higher than in other parts of the world. However, while the topic should have relevance for many, the detail provided is often simply too context specific making it difficult for an external reader to really see possible synergies.

 Overall, the description of the initiative is relatively well-described. Whether the description adds to what the original 12 tips article offered in terms of applying change models is possibly less clear. This might have been addressed by more overtly emphasizing links to existing literature (this is done in some instances, but more would have been useful), and by broadening/extending the discussion beyond the local. Other areas that could benefit from further explanation include detailing what the SWOT analysis added in terms of the ‘internal environment’. Could you provide some justification for the ‘various instructional strategies’ that were adopted and how did these differ from ‘traditional teaching methods’? What sort of evaluation was done to be able to be able to make the claim that ‘adopting the principles of project management greatly improved efficiency and effectiveness’?

 In some instances the text is unclear and the article could benefit from technical editing.

If evidence from practice is presented, are all the underlying source data available to ensure full reproducibility?

Yes

Is the topic of the practical tips discussed accurately in the context of the current literature

Partly

Are all factual statements correct and adequately supported by citations?

Partly

Are the conclusions drawn balanced and justified on the basis of the presented arguments?

Partly

Are arguments sufficiently supported by evidence from the published literature and/or the authors’ practice?

Partly

Reviewer Expertise:

In the context of this article: curriculum renewal

I confirm that I have read this submission and believe that I have an appropriate level of expertise to confirm that it is of an acceptable scientific standard, however I have significant reservations, as outlined above.

# Peer review: Reviewer response for version 1

**DOI:** 10.21956/mep.18782.r31898

**Published:** 2022-05-16

**Authors:** Naveed Yousuf, Tabassum Zehra

**Affiliations:** 1 Department for Educational Development, Faculty of Health Sciences, Aga Khan University, Karachi, Pakistan; 2 Aga Khan University, Karachi, Pakistan

The article presents a case study of successfully implementing a new Trauma Evaluation and Management course in an already established undergraduate medical education curriculum aligned to the national needs, institutional vision, and programmatic outcomes. It is a useful resource for academic leaders, faculty, and administrative staff involved in a curricular change.

 This is a good example showcasing how best practices from around the world can be successfully adopted in other regions if contextual factors are taken into consideration. In the present case, an international course on Trauma Management was adopted from well-developed western context and implemented in a developing country like Pakistan using recommended and evidence-based approaches. 

 The authors share their practical experience of successfully using the ‘Twelve tips for applying change models to curriculum design, development and delivery’ published by Judy McKimm & Paul Kneath Jones in 2018. The twelve tips facilitated the authors to undertake a holistic approach to curricular change covering various aspects ranging from identification of the core team, appropriate engagement with internal and external stakeholders, and resource management.

 We have the following suggestions which we believe will make the article more useful for readers:

1. Tip #1: Clearly specify the scope of curricular change in the beginning.
2. Tip #5: Include discussion on the internal environment and culture of the institution and how did it facilitate and/ or hindered the process. Also, what is meant by ‘All these facets need to have shared values’? This statement needs clarification and elaboration.
3. Tip #7: Mention the approach(es) for change that were instituted. The original article by McKim and Jones (2018) mentioned OIE (outcomes, interests, and emotions) approach.
4. Tip #8: Mentioning the structure or framework for project management employed will help readers to apply the same at their institutions.

Overall, this is a very comprehensive and interesting article presenting a successful case study of curricular change applying a structured framework. The lessons shared will greatly help readers in implementing curricular change at their institutions successfully.

If evidence from practice is presented, are all the underlying source data available to ensure full reproducibility?

Yes

Is the topic of the practical tips discussed accurately in the context of the current literature

Yes

Are all factual statements correct and adequately supported by citations?

Yes

Are the conclusions drawn balanced and justified on the basis of the presented arguments?

Yes

Are arguments sufficiently supported by evidence from the published literature and/or the authors’ practice?

Yes

Reviewer Expertise:

Medical Education, Curriculum Development, Assessment and Psychometrics, Academic Leadership

We confirm that we have read this submission and believe that we have an appropriate level of expertise to confirm that it is of an acceptable scientific standard.
